# Supplementary material for: Inter and transgenerational impact of H3K4 methylation in neuronal homeostasis
Source: Life Sci Alliance. 2023 May 24;6(8):e202301970. doi: 10.26508/lsa.202301970 (PMC10209521; doi:10.26508/lsa.202301970)
Supplement: Supplementary file 2 [file LSA-2023-01970_TableS2.docx]

**Supplementary Table 2: Neuroanatomical analysis**

**Neurons ctrl Heat F1 Heat F2 Heat F3 Starvation Maternal Age**

**AVK^A^**  0% 1%n.s 1%n.s 2%n.s 0%n.s 0%n.s

**PVP^A^**  7% 17%** 17%** 15%* 5%n.s 28%***

**PVQ^A^** 5% 16%* 27%**** 29%**** 9%n.s 24%****

**HSN^A^** 5% 15%** 14%** 17%**** 5%n.s 9%n.s

**HSN^B^** 4% 16%** 15%* 12%n.s 14%** 7%n.s

**VD&DD^C^** 28% 38%n 34%n.s. 28%n.s 31%n.s 24%n.s

**ALM^D^** 0% 0%n.s 1%n.s 0%n.s 0%n.s 0%n.s

**AVM^E^** 0% 1%n.s 1%n.s 0%n.s 0%n.s 0%n.s

**PLM^D^** 3% 5%n.s 5%n.s 2%n.s 7%n.s 4%n.s

**PVM^E^** 3% 8%n.s. 4%n.s 1%n.s 7%n.s 4%n.s

**^A^**Axons fail to respect the ventral midline and defasciculates

**^B^**Cell bodies fail to reach position at midbody before the vulva

**^C^**Axons extends commissures on the on wrong side of the animal

**^D^**Axons defasciculate and extend towards the ventral or dorsal nerve cord

**^E^**Cell body misplaced from its ventral sublateral position and/or axon extends posteriorly or anteriorly before reaching the ventral nerve cord
