# Supplementary material for: Inter and transgenerational impact of H3K4 methylation in neuronal homeostasis
Source: Life Sci Alliance. 2023 May 24;6(8):e202301970. doi: 10.26508/lsa.202301970 (PMC10209521; doi:10.26508/lsa.202301970)
Supplement: Supplementary file 3 [file LSA-2023-01970_TableS3.docx]

**Strain list**

**Strain Genotype times outcrossed**

N2 bristol wild type -

ZR1263 *oyIs14*[*sra-6p::GFP*] V 3

ZR1264 *hdIs26*[*odr-2p::CFP + sra-6::DsRed2*] III 3

ZR1057 *zdis5*[*mec-4p::GFP*] I 3

ZR1058 *oxIs12*[*unc-47p::GFP*] X 3

ZR1060 *zdIs13*[*tph-1p::GFP*] IV 3

ZR1064 *set-2(zr1208)*[*Y1397F-isoA*]*)* III 5

ZR1065 *set-2(zr1208)* III; *oyIs14* V -

ZR1166 *set-2(zr1208)* *hrde-1(tm1200)* III; *oyIs14* V -

ZR1124 *set-2(zr2012)*[*R1842W-isoA*]*)* III; *oyIs14* V 5

ZR1171 *hrde-1(tm1200)* III; *oyIs14* V -

ZR1084 *ash-2(tm1905)* II; *oyIs14* V 3

ZR75 *unc-6(ev400)* X; *oyIs14* V 1

ZR1265 *set-16(zr1804*[*H2410K*]*)* III 5

ZR1266 *set-16(zr1804)* III; *oyIs14* V -

ZR1161 *daf-2(e1370)* III; *oyIs14* V -

ZR1167 *daf-16(mu86)* I; *oyIs14* V -
